# 

**Published:** 1845-03

**Authors:** 


CONTENTS:

Rush Medical College.—Surgical Clinic. . . . . . . 177
Fatal Case of Visceral Disease, with Cerebral Symptoms—Post Mortem Examination. By J. Stickel, M.D., of Galena, Ill. 179
Practical Medicine, &c.
Bougies with Alum, . . . . 180
Croton Oil Plaster, . . . . 181
Bibliographical Notices.
Positive Theory of the Fecundation of the Mammiferæ. By F. A. Pouchet, M.D., Paris, 1842. 181
Proof of the Periodical Ripening and Separation of the Ova of Mammiferæ and Man, independent of Coitus, being the first condition of their Propogation. By Th. L. W. Bischoff, M.D., P. D., Giessen, 1844, . . 181
Our Journal, . . . . . 188
Index, . . . . . . 190

